# Prognosis in the Case of Granular Kidney

**Published:** 1906-01-06

**Authors:** 


### PROGNOSIS IN THE CASE OF GRANULAR KIDNEY.

In the course of a clinical lecture upon this subject Dr. Hale White [1] observes that the most valuable prognostic feature is the presence of albuminuric retinitis. But it is necessary to distinguish between two varieties of retinal change which are found in association with albuminuria. One of these is largely inflammatory, and is best shown by optic neuritis when present. This condition may occur in cases of acute Bright's disease, and may disppear as the patient recovers. The other variety is degenerative, and consists of bright white patches like mother of pearl let into the retina. This is due to fatty degeneration of nerve-fibres, and is of bad prognostic significance. It is doubtful whether patients exhibiting this variety of retinitis ever recover. In one series 85 per cent. of the patients presenting this form of albuminuric retinitis were dead within a year of the discovery of the retinitis, and a further 9 per cent. died in the course of the second year—*i.e.* 94 per cent. died within two years.

The prognostic element next in importance is the age of the patient, for young subjects with granular kidney seldom if ever live long. It is also to be remembered that while patients in later middle life who suffer from granular kidney generally die from cerebral hæmorrhage, pneumonia, or some other common associate of the disease, patients who develop granular kidney in early middle life almost invariably die of uræmia.

Great general weakness is an ominous sign. Pale, thin subjects stand granular kidney very badly, and, generally speaking, when anybody who suffers from this disease takes voluntarily to his bed, he has not long to live. Again, the dermatitis which is commonly associated with granular kidney is a sign of bad significance, for such patients seldom recover at all. Even when the dermatitis improves the end is not generally long postponed. Diarrhœa is a dangerous sign, that is to say, if it be severe or long continued, for then it commonly signifies colitis or enterocolitis.

Uræmia is a feature of importance. Although recovery may occasionally ensue upon states of coma which are regarded as hopeless, such a sequence is quite exceptional; when uræmic symptoms such as restlessness, insomnia, and bad headache last for a week or more the patient rarely recovers. It is advised, in passing, that such symptoms are best met by chloroform inhalations, repeated frequently if necessary. Cerebral hæmorrhage is of far less grave import than the symptoms above enumerated, but it is, of course, grave. In such a case if the coma does not begin to abate within twenty-four hours, the patient will almost certainly die: if he has Cheyne-Stokes' breathing as a consequence of cerebral hæmorrhage he will almost certainly die: if there is much mucus in the lungs he will probably die: if his paralysis is bilateral he will probably die, as also will he if his temperature is very low—(for this suggests a considerable loss of blood)—or very high—(for this indicates disorganisation of the thermotaxic mechanism). Further, apart from epistaxis, bleeding from other sites as well as from the cerebral vessel is a bad sign.

Pneumonia and pleurisy, peritonitis and pericarditis, all common complications of granular kidney, are all at the same time fatal ones as a rule.

As regards the information to be derived from the urine, the most important matter from the point of view of the prognosis is the observation whether or not the urine is being suppressed. If it is, the prognosis is bad in the extreme. A low specific gravity indicates that the kidneys are discharging few solids, and is therefore an indication of danger. Blood-casts, granular and epithelial casts all indicate by their presence in the urine that pathological processes are active in the kidney, and augur badly. Hyaline casts are probably of little significance, at all events too little is known of their significance to admit of reliable inference. The amount of albumin in the urine is of little prognostic value, although a steady increase in the quantity indicates that tubal nephritis is supervening, while if the latter be previously present, a diminution in the quantity of albumin indicates that the tubal inflammation is subsiding. Finally, much information of value is to be gathered from a careful examination of the cardio-vascular system. A thick artery, with an exaggerated tension shows that the patient is in danger of cerebral hæmorrhage.

The lecturer, in conclusion, urged his audience not to rely upon their fingers for estimates of tension in the pulse, on account of the impossibility under such circumstances of making any accurate comparison of tensions at intervals of time of any length. He recommended the use of an instrument described by Dr. C. J. Martin in the *British Medical Journal*, 1905, vol. 1, p. 865.

[1] Clin. Jour., December 6, 1905.

